# [Corrigendum] T7 peptide inhibits angiogenesis via downregulation of angiopoietin‑2 and autophagy

**DOI:** 10.3892/or.2024.8779

**Published:** 2024-07-17

**Authors:** Fuhai Wang, Xiaofeng Dong, Peng Xiu, Jingtao Zhong, Honglong Wei, Zongzhen Xu, Tao Li, Feng Liu, Xueying Sun, Jie Li

Oncol Rep 33: 675–684, 2015; DOI: 10.3892/or.2014.3653

Following the publication of this article, an interested reader drew to the authors' attention that two pairs of protein bands featured in the western blots in Fig. 3A and 5D on p. 679 and 681 respectively appeared to be strikingly similar. After having re-examined their original data, the authors realized that Fig. 5D had been assembled incorrectly.

The revised version of Fig. 5, now including the correct data for Fig. 5D, is shown on the next page. Note that the errors made in terms of assembling the data in Fig. 5 did not greatly affect either the results or the conclusions reported in this paper, and all the authors agree to the publication of this corrigendum. The authors regret that these errors went unnoticed prior to the publication of their article, are grateful to the Editor of *Oncology Reports* for allowing them this opportunity to publish this corrigendum. They also apologize to the readership for any inconvenience caused.

## Figures and Tables

**Figure 5.**
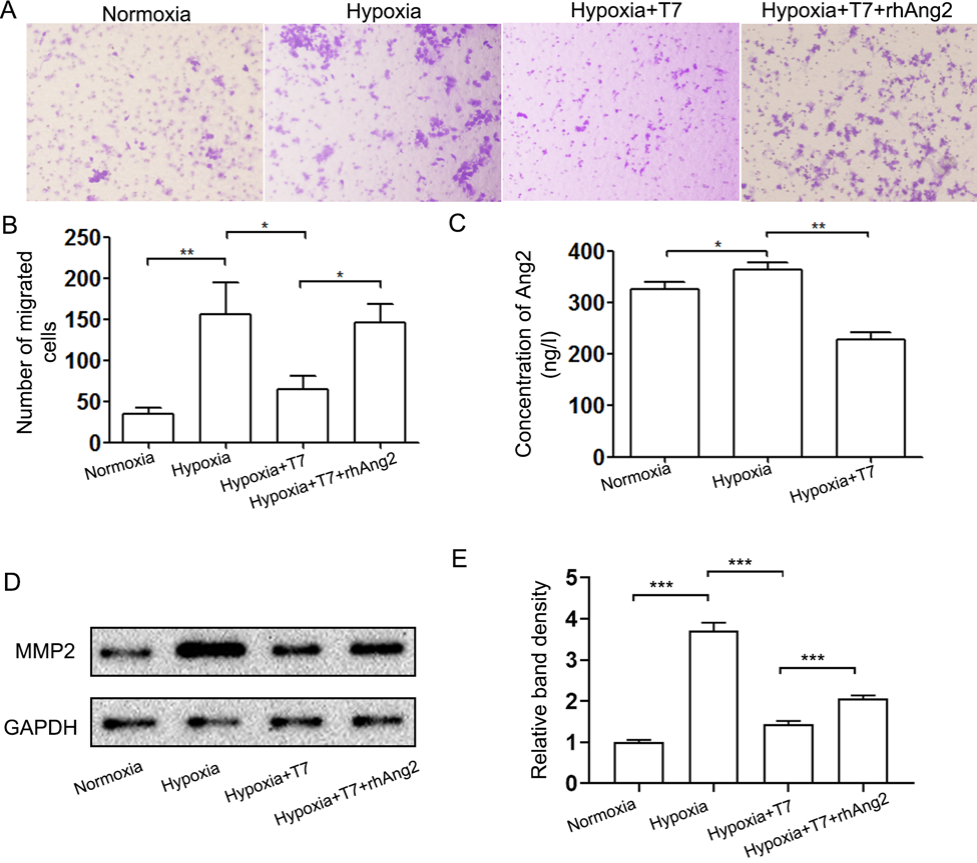
T7 peptide inhibits the invasion of HepG2 cells by inhibition of Ang2 expression. (A) Transwell plates were used to analyze the effect of the T7 peptide and Ang2 on the invasion of HepG2 cells (magnification, ×100). (B) The number of migrated cells was counted from 5 random views. (C) ELISA kit was used to detect the concentrations of Ang2 in supernatants of the human umbilical vein endothelial cell (HUVEC) culture. (D) Expression of MMP-2 was analyzed by western blotting. (E) The density of each band was quantified and normalized to that of GADPH. *P<0.05, **P<0.01.

